# Add‐on effect of PSORI‐CM01 to topical calcipotriol for moderate psoriasis vulgaris: A multi‐center, randomized, double‐blind pilot study

**DOI:** 10.1002/ctm2.286

**Published:** 2021-01-15

**Authors:** Shefton Parker, Anthony Lin Zhang, Claire Shuiqing Zhang, Greg Goodman, Zehuai Wen, Yuhong Yan, Danni Yao, Huimei Wu, Hao Deng, Chuanjian Lu, Charlie Changli Xue

**Affiliations:** ^1^ China‐Australia International Research Centre for Chinese Medicine School of Health and Biomedical Sciences RMIT University Bundoora Victoria Australia; ^2^ The Dermatological Institute of Victoria South Yarra Victoria Australia; ^3^ Guangdong Provincial Hospital of Chinese Medicine Guangzhou China; ^4^ Guangdong Provincial Academy of Chinese Medical Sciences Guangzhou China; ^5^ The Second Clinical College Guangzhou University of Chinese Medicine Guangzhou China

**Keywords:** calcipotriol, Chinese herbal medicine, clinical trial, pilot, placebo, psoriasis

## Abstract

**Background:**

Mild‐moderate psoriasis vulgaris is a common dermatological autoimmune condition with limited conventional therapeutic options. Safe and effective adjunct therapies to topical non‐steroidal antipsoriatic therapy are needed. The oral Chinese herbal medicine (CHM) formula PSORI‐CM01 has been evidenced potential antipsoriatic pharmacological activity. This article reports a pilot study which was designed as a double‐blinded, placebo‐controlled randomized controlled trial (RCT) evaluating the effects of PSORI‐CM01 when added to topical calcipotriol cream.

**Methods:**

People with moderate psoriasis vulgaris were randomized to receive oral PSORI‐CM01 or placebo administered for 12 weeks in combination with calcipotriol. The primary clinical outcome was the change of psoriasis area severity index (PASI) score at week 12 and week 24. Secondary clinical outcomes were PASI75, PASI50, relapse rate, change in body surface area, dermatology life quality index and Skindex29, and adverse events (AEs). Participants’ satisfaction and willingness to repeat were also assessed.

**Results:**

The pilot study was conducted in Australia and China, 29 participants were randomized with 26 completed the treatment and follow‐up. Participants’ baseline basic characteristics were comparable. No between‐group statistical significance was found on pre‐defined clinical outcome measures, although there seemed a trend of treatment effects favoring the combination of PSORI‐CM01 with calcipotriol. Frequency and severity of AEs were similar between two groups, with no severe AEs reported.

**Conclusions:**

The design and duration of the study appears feasible. A proper powered RCT with slight adjustments in the methods is needed to reveal the add‐on effects of oral CHM PSORI‐CM01. The experience and results from this pilot study will contribute to the refine of objectives and design of a future study, and assist the sample size calculation for the full‐scale RCT.

## 1 INTRODUCTION

Psoriasis vulgaris is a chronic, recurring, multisystem inflammatory disease, commonly presenting with well‐demarcated red plaques with silvery scaling that can occur on any part of the skin, including the nails, palms, soles, and genitalia, with the most commonly seen areas being elbows, knees, and scalp.^1^ Psoriasis vulgaris is a non‐communicable disease, plaque areas can have severe pruritus, which result in pinpoint bleeding (Auspitz's sign) upon scratching.^2^ The precise causes of psoriasis remain unknown but it is believed to be related to immune‐regulation with genetic and environmental contributions.^1^, ^3^ People with psoriasis experience physical discomfort, and the visual appearance of plaques can cause embarrassment as well, both of them significantly impact quality of life (QoL).^4^

Conventional therapies for psoriasis target immune response, reducing inflammation and keratinocyte proliferation. The German evidence‐ and consensus‐based (S3) guideline for the treatment of psoriasis states that there is no clear treatment protocol as cases must be evaluated on an individual basis.^5^ The key recommendations for basic therapy involve one or a combination of topical and phototherapy/systemic therapy.^5^ For moderate‐to‐severe cases, the use of biologics, such as TNF‐α inhibitor, IL‐17 antibody, and IL‐12/IL‐23 antagonists, has become a welcome addition in psoriasis management.^1^ The National Institute for Health and Care Excellence (NICE) recommends topical therapy on top of emollients as the first line treatment for psoriasis vulgaris.^6^ Clinical guidelines also mentioned the limitations of corticosteroids, including causing psoriasis to become unstable, causing irreversible skin atrophy, or systemic side effects upon long‐term or extensive use. Subsequently, they advised against long‐term use of corticosteroid by suggesting a break after 4 weeks and replacement with non‐steroid‐based topical therapies such as vitamin D, vitamin D analogues (e.g., calcipotriol), or coal tar.^6^

Patients with psoriasis frequently seek complementary and alternative medicine treatments due to the insufficient treatment effects or unwanted side effects of conventional medications.^7^ Previous meta‐analyses of randomized controlled trials (RCTs) indicated that orally administered Chinese herbal medicine (CHM) was safe and effective for psoriasis when compared to placebo,^8^ and that the combination of CHM and conventional therapy was more effective than conventional therapy alone.^9^

A well‐known Chinese medicine master Guo‐Wei Xuan developed a CHM formulation (Yin Xie Ling) for the management of psoriasis. Yin Xie Ling has been used for a few decades in clinical practice and shown promising effectiveness for psoriasis.^10^, ^11^, ^12^, ^13^, ^14^ PSORI‐CM01 is an optimized version of Yin Xie Ling containing seven herbs.^10^, ^11^, ^12^, ^13^, ^14^ Ultra‐high liquid chromatography mass spectrometry has determined 14 marker compounds in the formula, assisting batch‐to‐batch consistency and quality control.^12^ The chemical composition of PSORI‐CM01 consists predominantly of organic acids, phenolic acids, flavonoids, and terpenoids.^12^ It has been identified that the therapeutic anti‐inflammatory mechanism of PSORI‐CM01 is via inhibition of NF‐kB p65 expression.^13^ In psoriatic murine model, PSORI‐CM01 inhibits epidermis hyperplasia, reduces expression of cyclin B2, and inhibits HaCaT cell proliferation. In HaCaT psoriasis model, PSORI‐CM01 reduces IFN‐γ‐induced mRNA expression of inflammatory cytokines such as IL‐6, IL‐12, and CXCL‐10.^13^, ^14^ The efficacy and safety of CHM formula PSORI‐CM01 as an add‐on therapy to conventional therapy has yet to be evaluated by clinical research.

We designed a double‐blind, randomized, placebo‐controlled, pilot clinical trial to evaluate the add‐on effects of this oral CHM formula, PSORI‐CM01 (named YXBCM01 in the published protocol), to topical calcipotriol.^15^ Here, we report the findings of the pilot study, discuss the practicality of the protocol, and investigate if the efficacy and safety of the interventions show a trend warranting a large‐scale RCT.

## 2 METHODS

This pilot study is designed as a randomized, double‐blinded, placebo‐controlled trial comparing the combination of oral CHM PSORI‐CM01 with topical calcipotriol to placebo plus topical calcipotriol. The trial protocol has been previously published and has received ethics approval from the RMIT University Human Research Ethics Committee and the Ethics Committee of Guangdong Provincial Hospital of Chinese Medicine.^15^ The study was prospectively registered with the Australian New Zealand Clinical Trials Registry on 12 May 2014 (ACTRN12614000493640).

### 2.1 Inclusion and exclusion criteria

Participants, who met the following inclusion criteria, were considered eligible:

- Aged between 18 and 70 years.
- Suffered from psoriasis vulgaris symptoms for at least 12 months.
- With psoriasis area severity index (PASI) score assessed as between 7 and 12.

Participants, who had any condition as follows, were not eligible:

- Using systemic drugs or phototherapy for psoriasis within 4 weeks of screening, or using other topical drugs for psoriasis within 2 weeks of screening.
- Other severe systemic disorders.
- Known abnormal liver or kidney function.
- Pregnant or breast‐feeding women.
- Unwilling or unable to cease other topical and systemic psoriasis‐related medication for the duration of the trial.

### 2.2 Participant recruitment

Participants were attracted from newspapers, online advertisements, posters displayed around RMIT University campuses, and from the outpatients’ department of dermatology at Guangzhou Hospital of Chinese Medicine outpatients. Further trial information was provided to interested persons by email, phone, or face to face, followed by a screening appointment for their eligibility. Written informed consent was obtained from all participants.

### 2.3 Participant involvement

Participants will be provided with a lay summary of the results when published. Treatment burden was assessed through participant responses to the acceptability and willingness of participants to repeat their interventions. Participants were not involved with the development of the study design.

### 2.4 Randomization and masking

Block randomization was prepared by an independent researcher using computer‐generated randomized allocation. Participants had a 1:1 chance of being randomized to PSORI‐CM01 plus calcipotriol or placebo plus calcipotriol. Randomization codes were individually sealed in opaque envelopes. Following the baseline assessment, the envelopes were opened sequentially for each participant by a research assistant who was not involved in the preparation of randomization codes. A contained code corresponded with a pre‐prepared package of study drugs. Throughout the study, the medicine dispensers, participants, outcome assessors, and members of the research team were all blinded to group allocation. Blinded data were unmasked upon the completion of statistical analyses.

### 2.5 Interventions

***Calcipotriol*** : This is a 1st line therapy for moderate psoriasis vulgaris commonly prescribed in cream or lotion form.^16^, ^17^ Calcipotriol (supplied as Daivonex) cream was dispensed in 30 g tubes (0.005% (50 μg/g)). Participants were instructed to apply the calcipotriol cream daily on affected body surface areas for 12 weeks (maximum) or until the complete clearance of lesions. In accordance with guidelines, dosages were determined at week 0 and week 6 by researchers who were unaware of participants’ group allocation (1% surface area coverage = 0.5 fingertip units),^1^ Between assessments, participants could increase or decrease its dosage along with the change of psoriasis severity.

***Oral CHM PSORI‐CM01*** : The PSORI‐CM01 consisted of botanicals Radix *Paeonia veitchii* Lynch (*chi shao*), *Sarcandra glabra* (*zhong jie feng*), *Rhizoma smilacis glabrae* (*tu fu ling*), *Curcuma zedoary (e zhu)*, *Fructus mume* (*wu mei*), *Radix arnebias* (*zi cao*), and *Radix glycyrrhizas* (*gan cao*).^12^, ^15^

***Placebo*** : Placebo granules were prepared from starch and contained no active ingredients. Placebo was packaged identically and matched as closely as possible to appearance, smell, and taste of dissolved and unprepared PSORI‐CM01. Dosage and administration instructions were identical to PSORI‐CM01.

Both PSORI‐CM01 and its placebo were prepared by Tianjiang Pharmaceutical Co. Ltd. (Jiangyin, Jiangsu Province, China) holding a good manufacturing practice certificate. Single‐dose sachets of PSORI‐CM01 or placebo granules were packaged weighing 5.5g. One sachet was dissolved in warm water and orally consumed after meals mornings and evenings for 12 weeks.

### 2.6 Outcome measures

The general evaluation of trial methodology was considered through observing the suitability of screening criteria, outcome measures for data collection, and participants’ compliance during the treatment and follow‐up phases.

In terms of the potential treatment effects of adding oral CHM to calcipotriol, the primary outcomes for assessing potential intervention efficacy was the change of PASI score from baseline (week 0) to end of treatment (week 12), and end of follow‐up phase (week 24). Other outcome measures for potential efficacy, participants’ satisfaction, and monitoring safety of the interventions are:

- Number of participants achieving 75% PASI improvement (PASI75) at week 12.^18^
- Number of participants achieving 50% PASI improvement (PASI50) at week 12.^18^
- Rate of lesions returning to 50% of baseline PASI score (relapse rate) at week 24.
- Change in body surface area (BSA).^19^
- Change in Dermatology life quality index (DLQI) score.^20^
- Change in Skindex29 score.^21^
- Acceptability and willingness to repeat the CHM treatment (Likert scale: definitely not, probably not, probably yes, definitely yes or unsure).
- Safety based on kidney and liver blood toxicology and monitoring of adverse events (AEs).

Outcomes related to psoriasis severity were assessed at recruitment, baseline, middle (week 6) and the end of treatment phase (week 12), and the end of follow‐up phase (week 24). AEs were monitored during the treatment and follow‐up phases, with blood tests being conducted at week 6, week 12, and week 24.

### 2.7 Trial procedures

**FIGURE 1:**
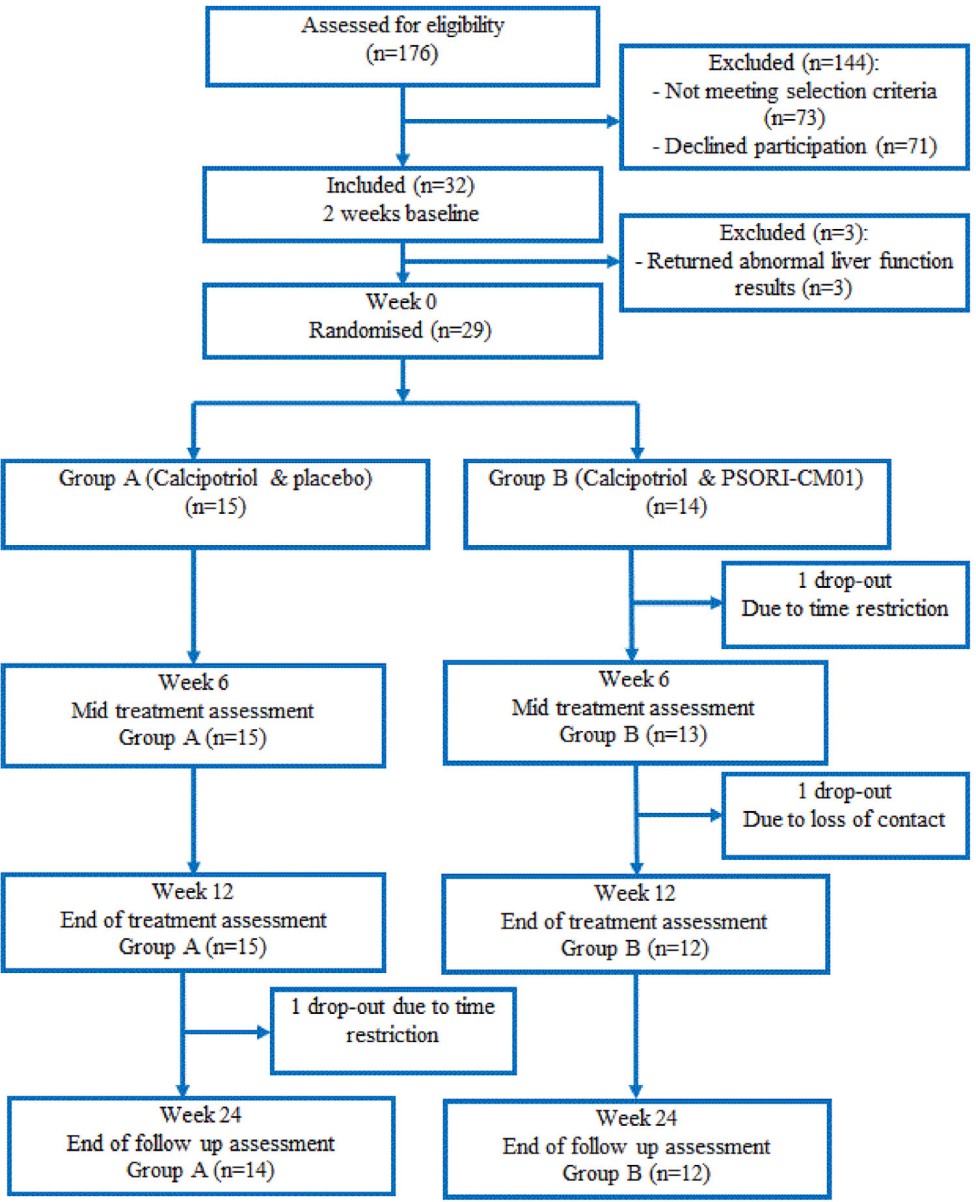
Flow diagram of trial procedure

Complete trial procedures have been previously published, and the pilot trial was conducted following these procedures.^15^ The trial procedure is shown in the flowchart (Figure. 1).

All participants completed a 2‐week run‐in phase, avoiding psoriasis‐related medications. Following the run‐in phase, participants attended a baseline assessment. Eligible participants were randomized and dispensed with study medications accordingly. Assessment of outcome measures was conducted in accordance with the previously published protocol. During the treatment phase, participants were required to complete case report forms (CRFs) to record their symptoms, medication usage, and AEs. An assessor who was blinded to group allocation collected psoriasis symptoms and AE information via fortnightly telephone follow‐up. In addition, three face‐to‐face assessments were conducted: a mid‐treatment assessment (week 6), an end‐treatment assessment (week 12), and final assessment at the end of non‐treatment follow‐up period (week 24). In these face‐to‐face assessments, all clinical outcomes including symptoms, AEs, and blood tests were repeated, with the same clinician performed clinical assessment to ensure consistency. An independent data safety monitoring board monitored safety and data processing.

### 2.8 Data management

Participants were required to record daily and weekly data in CRFs. Data collection was conducted via face‐to‐face and telephone interviews or assessments. All data were entered into a pre‐designed, password protected dataset by personnel blinded to group allocation. Data entry was performed continuously throughout the study using a double‐check method, with any correction or changes of written data in participants’ CRFs documented and dated.

### 2.9 Statistical analysis

The Statistical Package for the Social Sciences software version 21.0 for Windows (SPSS Inc., Armonk, NY) was used for data analysis. For the clinical outcomes, an intention‐to‐treat (ITT) analysis was used with missing values replaced using the last observation carried forward approach. Equivalence analyses were conducted of baseline characteristics and categorical data of both groups, using χ^2^ test for pairwise comparison of categorical variables and *t*‐tests for equivalence between continuous variables. Mean, standard deviation (SD), mean difference (MD), and 95% confidence interval (CI) were calculated for continuous data. For dichotomous and categorical data, risk ratio (RR) with 95% CI and χ^2^ test were used as appropriate. Outcomes were assessed for equivalence using χ^2^ test or *t*‐tests between the two groups at each assessment time point, with *P* < .05 difference considered statistically significant.

## 3 RESULTS

### 3.1 Trial recruitment and compliance

This pilot study was conducted between January 2015 and May 2016 in Australia, at the Research Hub of the School of Health and Biomedical Science at RMIT University and in China at the Guangdong Provincial Hospital of Chinese Medicine.

A total of 176 people were screened for inclusion, with 144 excluded. Of those excluded, 73 patients did not meet the inclusion criteria, and 71 declined participation. The predominant reason for declining participation was an inability to fulfil the study requirements.

Thirty‐two participants commenced the run‐in phase. Three participants were excluded during the run‐in phase due to abnormal liver function shown in blood test which did not meet the inclusion criteria. The remaining 29 participants were randomized into two groups: calcipotriol plus placebo (n = 15) and calcipotriol plus PSORI‐CM01 (n = 14). Eleven participants were recruited from the Australia site while 18 were from the China site. There were two dropouts from the PSORI‐CM01 group during the treatment phase, one due to time restrictions and the other lost to follow‐up. There was one drop‐out from the placebo group during the follow‐up phase (Figure 1).

### 3.2 Baseline characteristics

There were no other baseline characteristic imbalances except for the body mass index of participants was higher in the PSORI‐CM01 group compared to the placebo group (MD: −2.47, 95% CI [−4.68, −0.24]). Symptom measures such as the PASI score, BSA, QoL measures including the DLQI and Skindex‐29 were comparable at baseline (Table 1).

**TABLE 1:** Baseline characteristics

Characteristic and clinical outcomes		Group A (*n* = 15): Mean (SD) or numbers	Group B (*n* = 14): Mean (SD) or numbers
Age (years)		42.13 (15.08)	41.78 (11.85)
Age at onset of psoriasis (years)		26.4 (14.96)	23.86 (10.63)
Duration of psoriasis (years)		15.73 (11.90)	17.92 (10.55)
Weight (kg)		67.83 (9.45)	72.59 (11.84)
Body mass index^*^		22.87 (2.80)	25.33 (3.03)
Gender (male/female)		12/3	10/4
History of smoking (yes/no)		8/7	4/10
Previous use of CHM (yes/no)		10/5	7/7
Has family history of psoriasis (yes/no)		1/14	4/10
PASI		9.13 (1.54)	8.49 (1.80)
BSA		10.09 (3.35)	11.55 (6.38)
DLQI		10.27 (6.68)	6.93 (4.60)
Skindex 29	Symptoms	46.19 (15.24)	40.05 (22.46)
	Emotions	48.50 (20.89)	37.14 (21.99)
	Functioning	42.78 (18.51)	28.14 (22.87)
	Total score	50.25 (19.39)	37.90 (20.62)

Note: Group A, calcipotriol plus placebo; Group B, calcipotriol plus PSORI‐CM01.

^*^Data of baseline characteristics and outcome measures were comparable except for the body mass index.

Abbreviations: BSA, body surface area; DLQI, dermatology life quality index; PASI, psoriasis area and severity index; SD, standard deviation.

### 3.3 Clinical outcomes

**FIGURE 2:**
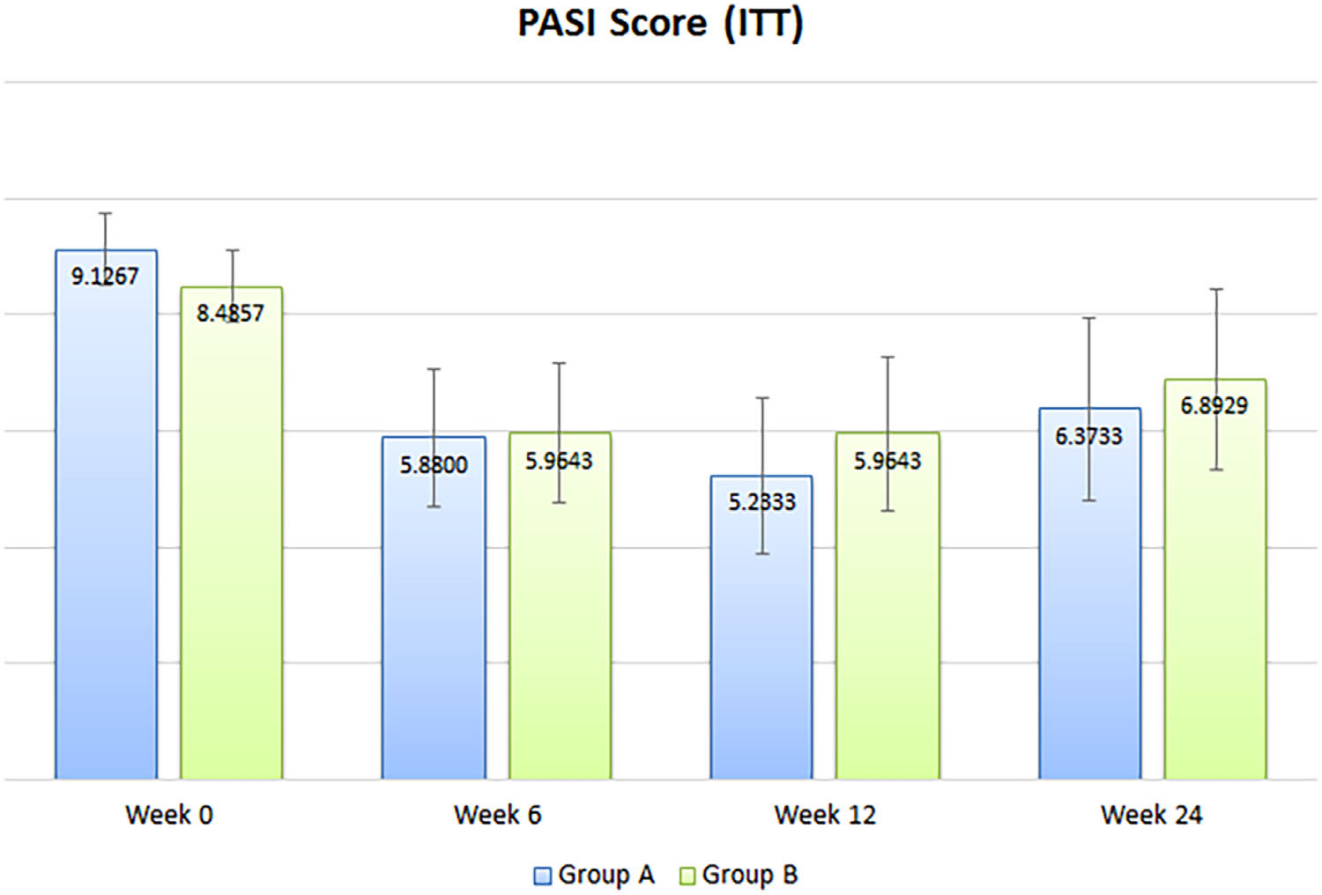
PASI score (ITT results)

Based on the ITT analysis, the reduction in PASI scores was greatest in the first 6 weeks (PSORI‐CM01: −3.16 versus placebo: −3.25). At week 24, the PASI scores in both groups increased from week 12, but with no statistical difference between groups (Figure 2). There was no significant difference between groups in the changes in PASI, BSA, DLQI, and Skindex‐29 scores in week 12 and week 24, respectively (Table 2). Sensitivity analysis using non‐imputed data showed no changes in the results.

**TABLE 2:** Main outcome measures (intention‐to‐treat)

Outcomes measure		Change score from week 0 to week 12			Change score from week 12 to week 24		
		Group A (n = 15): Mean (SD)	Group B (n = 14): Mean (SD)	MD (95%CI)	Group A (n = 15): Mean (SD)	Group B (n = 14): Mean (SD)	MD (95% CI)
PASI		−3.89 (2.29)	−2.52 (3.89)	−1.37 (‐3.86, 1.12)	1.14 (3.57)	0.93 (2.59)	0.21 (−2.18, 2.61)
BSA		−2.37 (4.93)	−3.16 (4.10)	0.79 (−2.67, 4.26)	1.17 (4.51)	0.84 (3.62)	0.34 (−2.74, 3.43)
DLQI		0.80 (5.52)	−1.57 (4.72)	2.37 (−1.55, 6.29)	−1.47 (5.66)	−0.50 (3.72)	−0.99 (−4.64, 2.71)
Skindex 29	Symptoms	−9.52 (17.94)	−3.53 (17.06)	−5.99 (−19.36, 7.37)	2.62 (19.67)	−4.63 (9.07)	7.25 (−4.57, 19.07)
	Emotions	−0.33 (12.60)	−4.29 (15.92)	3.95 (−6.95, 14.85)	−1.88 (12.39)	−0.54 (12.73)	−1.35 (−10.92, 8.23)
	Functioning	−1.15 (13.14)	−7.30 (15.87)	6.15 (−4.92, 17.22)	−1.31 (12.86)	−1.43 (7.46)	−0.12 (−7.97, 8.21)
	Total score	−4.47 (15.49)	−6.25 (15.66)	−1.77 (−10.10, 13.65)	0.13 (15.14)	−2.34 (7.30)	2.46 (−6.70, 11.63)

Note: Week 0, baseline; week 12, end of treatment phase; week 24, end of follow‐up phase; group A, calcipotriol plus placebo; group B, calcipotriol plus PSORI‐CM01.

Abbreviations: BSA, body surface area; CI, confidence interval; DLQI, dermatology life quality index; MD, mean difference; PASI, psoriasis area and severity index; SD, standard deviation.

At week 12, three participants (21%) achieved PASI75, and six participants (43%) achieved PASI50 clearance from the PSORI‐CM01 group. Comparatively, only seven participants (47%) achieved PASI50 clearance at week 12 in the placebo group. At week 24, there were two relapses in the PSORI‐CM01 group (14%) and three in the placebo group (20%) (Table 3).

**TABLE 3:** PASI75, PASI50, relapse rate, satisfaction, willingness to repeat treatment (intention‐to‐treat)

Outcomes	Group A: (n = 15)	Group B:(n = 14)	Significance	
PASI75 (week 12) yes/no	0/15	3/11	χ^2^ = 3.59	*P =* .06
PASI50 (week 12) yes/no	7/8	6/8	χ^2^ = 0.04	*P =* .84
Relapse rate (week 24) (relapse/no relapse/did not achieve PASI50 at end of treatment)	3/4/8	2/4/8	χ^2^ = 0.17	*P =* .92
Willingness to repeat treatment (week 12) yes/no/unsure	8/2/5	5/5/4	χ2 = 2.01	*P* = .36
Willingness to repeat treatment (week 24) yes/no/unsure	10/3/2	7/4/3	χ2 = 0.84	*P* = .66
Satisfaction (0‐10): (week 12) mean (SD)	7.80 (3.10)	5.14 (3.66)	*t* = 2.12	*P* = .04^*^
Satisfaction (0‐10): (week 24) mean (SD)	6.73 (2.66)	6.57 (3.76)	t = 0.13	*P* = .89

Note: Week 12, end of treatment phase; week 24, end of follow‐up phase; group A, calcipotriol plus placebo; group B, calcipotriol plus PSORI‐CM01; PASI, psoriasis area and severity index.

^*^Statistically significant.

It is worth noting that at week 12, the Skindex‐29 function domain score favored PSORI‐CM01 (20.83 ± 16.93) compared to placebo group (42.43 ± 20.09) (MD: −19.63, 95% CI [−5.42, −33.85]), but the change score was not significantly different between two groups. Similarly, at week 24, the Skindex‐29 function domain score and total score favored the PSORI‐CM01 group (MD: −19.75, 95% CI [−4.95, −34.55] and MD: −16.17, 95% CI [−1.84, −30.50] respectively), but the change score from week 12 to week 24 was not significantly different between the two groups (Table 2). In addition, there was significant difference in DLQI scores between two groups at week 12 (MD: −5.71, 95% CI [−0.64, −10.78]) and week 24 (MD: −4.74, 95% CI [−1.24, −8.24]), however, the change in DLQI scores between groups was not significantly different at both timepoints.

### 3.4 Participants’ satisfaction and willingness to repeat

Based on the ITT analysis, there was no significant difference between groups in participants’ willingness to repeat treatment at week 12 (*χ* ^2^ = 2.01, *P* = .36) and at week 24 (*χ* ^2^ = 0.84, *P* = .66) (Table 3). Participants’ satisfaction scores favored the placebo group (PSORI‐CM01: 5.14 ± 3.66 versus placebo: 7.80 ± 3.10) at week 12 but there was no difference at week 24 (PSORI‐CM01: 6.57 ± 3.76 versus placebo: 6.73 ± 2.66) (Table 3).

### 3.5 Safety

No dropouts due to AEs were reported. During the treatment phase, 19 AEs were reported by the PSORI‐CM01 group and 18 by the placebo group (Table 4). Seven (46.7%) people in the PSORI‐CM01 group and nine (60%) in the placebo group reported at least one AE (*P* = .61). Mild diarrhea (n = 5) or nausea (n = 5) were the most commonly reported AEs in both groups during the treatment phase. Both groups had one event of lethargy, self‐reported as severe, but did not require any medical management. One case of itch required follow‐up with a medical practitioner but did not need any treatment, and the itch did not persist (Table 4).

**TABLE 4:** Adverse events

	Treatment phase (week 0‐12)		Follow‐up phase (week 12‐24)	
Adverse events	Group A (n = 15)	Group B (n = 14)	Group A (n = 14)	Group B (n = 12)
Anxiety	1‐mild	0	0	0
Diarrhoea	2‐mild 1‐unclear	2‐moderate	0	0
Dysuria	1‐unclear	0	0	0
Dizziness	1‐mild	1‐mild	0	0
Erythema	1‐mild	1‐mild	0	0
Fever	0	1‐moderate	0	0
Headache	1‐moderate	1‐mild	1‐moderate	0
Itchiness	1‐moderate 1‐severe	1‐mild 2‐moderate	0	0
Lethargy	1‐moderate 1‐severe	1‐mild 1‐severe	0	0
Nausea	4‐mild	1‐mild	0	0
Nasal discharge	0	2‐ mild	0	0
Periodontitis	0	0	1‐moderate	0
Pigmentation	0	0	1‐mild	0
Polyuria	2‐unreported	0	0	0
Respiratory tract infection	0	0	0	1‐moderate
Skin irritation	0	2‐moderate	0	1‐moderate
Tinnitus	2‐unreported	0	1‐mild	0
Thirst	0	2‐mild	0	0
All adverse events reported	19	18	4	2
Number of participants reported adverse events (%)	7 (46.7%)	9 (60%)	3 (21.4%)	1 (8.3%)
χ2 (*P*‐value)	0.26 (.61)		1.01 (.32)	

Note: Week 0, baseline; week 12, end of treatment phase; week 24, end of follow‐up phase; group A, calcipotriol plus placebo; group B, calcipotriol plus PSORI‐CM01.

During the follow‐up phase, four AEs were reported in the placebo group and two in the PSORI‐CM01 group. Three people in the placebo group (21.4%) and one in the PSORI‐CM01 group (8.3%) reported at least one AE (Table 4).

## 4 DISCUSSION

This article reports the results of a pilot trial of a double‐blinded, placebo‐controlled RCT. The practicality of the RCT design was tested, and the efficacy results obtained in the pilot study can be used for calculating the sample size of a full‐scale RCT. Conducting this pilot trial in two recruitment sites (Melbourne, Australia and Guangzhou, China) provides evidence of the possibility for future trial planning with international recruitment. It should be pointed out that the initial design of the pilot study was aiming at 30 participants, as stated in the published protocol.^15^ However, due to the low rate of meeting the selection criteria, the recruitment was rather slow. The recruitment was ceased when 29 participants were recruited to ensure all participants could complete the treatment phase before the expire date of the trial medication (CHM and placebo). It is known that a general flat rule of a pilot study is to "use at least 30 subjects or greater to estimate a parameter",^22^ whereas Julious suggested a minimum sample size of 12 subjects per treatment arm.^23^ Based on these recommendations, we consider a sample size of 29 should be sufficient for this pilot study.

Participants who entered this pilot study showed acceptable compliance, with only three (10.3%) dropouts during the entire trial, due to time restriction or loss of contact. The findings suggest that the design of this study consisting a 12‐week CHM treatment phase, a 12‐week follow‐up phase, and three face‐to‐face assessment is feasible, and the protocol can be executed.

The results on participants’ satisfaction with the treatment favored the placebo group at the end of treatment phase, but there was no between‐group difference at the end of follow‐up phase. On the other hand, the "willingness to repeat the treatment" assessment showed equivalent results at both the end of treatment and follow‐up phases. The less satisfaction of CHM treatment could be caused by the unpleasant taste of herbal medicine, which is commonly seen in CHM clinical practice. Nevertheless, at the end of the study, results of these two outcomes showed no between‐group difference, suggesting the CHM intervention was not too burdensome. However, the small sample size of this pilot study limits our certainty on a conclusion. It is worth considering re‐assess these outcomes in future study to provide more evidence for clinical application.

The clinical outcomes may provide an indication of efficacy, but as a pilot trial, the sample size is too small to allow for sufficient evaluation for conclusive results. These outcomes can, however, assist in the effect size calculation for a full‐scale trial. The symptom outcomes from this pilot study showed an overall improvement in both groups by week 12, with only the PSORI‐CM01 group achieving PASI75. However, there was not statistically significant between‐group difference detected for the change scores of all clinical outcome measures. On the other hand, QoL outcome measures showed that the add‐on effects of PSORI‐CM01 were significantly greater than placebo, indicating that QoL outcomes should be taken into consideration when determining a sample size with adequate power.

With regard to the evaluation of the safety of the interventions, this study showed that both groups had similar occurrences of AEs. This is in agreement to the findings of a previous systematic review on AEs in herbal drug studies.^24^ The reported AEs were predominantly mild or moderate, with some of these unlikely related to the interventions. There were no severe AEs, and none of the AEs resulted in dropouts. This suggests that the treatment regime involving 12 weeks oral administration of PSORI‐CM01 combined with topical calcipotriol is safe and well‐tolerated.

In this study, the selected conventional treatment was the vitamin D analogue, calcipotriol. Vitamin D analogues are included in the first line topical therapy for psoriasis.^5^, ^6^ Their main mechanism of action is related to inflammatory suppression,^25^, ^26^ keratinocyte proliferation inhibition, and increased differentiation.^5^, ^27^ Calcipotriol (also known as calcipotriene) was the first vitamin D analogue to be approved for the treatment of psoriasis vulgaris.^27^ A systematic review on topical therapies for psoriasis suggested that calcipotriol was more superior than dithranol, coal tar, and other vitamin D3 analogues.^28^ While it has been shown that a combination topical therapy of calcipotriol with the corticosteroid betamethasone is better than either drug administered alone,^5^, ^28^ based on the NICE guidelines on safe use of corticosteroids, very potent and potent corticosteroids should not be used for more than 4 and 8 consecutive weeks, respectively.^6^ Protocols of previous clinical studies using corticosteroids seem to abide by this recommendation, ensuring that the treatment duration with corticosteroids was never longer than 8 consecutive weeks.^5^, ^28^ Considering the 12‐week treatment duration in our trial protocol design and weighing out the risk‐benefit ratio, calcipotriol without corticosteroids appeared to be a more suitable representative conventional therapy for our study.

One of the limitations identified during this pilot study is the high number of participant exclusions and declined participation during screening. Of the participants who did not meet the inclusion criteria, the PASI ≥7 was the main barrier. It was noted that some persons who were assessed with a PASI score less than 7 still reported considerable burden from psoriasis to their QoL. PASI has been reported to lack sensitivity to change in milder versions of psoriasis,^29^ which may also explain the similar PASI scores in the outcomes of this study. Adjusting the inclusion criteria by lowering the PASI score for inclusion or adopting a more sensitive outcome measure, such as the PrecisePASI,^30^ would allow the evaluation of the interventions on a broader population suffering from mild psoriasis. Broadening the inclusion criteria could also further assist in achieving recruitment number targets.

## 5 CONCLUSION

This pilot study shows that the design and duration of the study appears to be practical, however, slightly adjusting the selection criteria prior to conducting the full‐scale RCT may improve participants’ recruitment. Pre‐defined clinical outcome measures did not detect any statistical significance, although there seemed a trend of treatment effects favoring the combination of PSORI‐CM01 with calcipotriol. A proper powered RCT is needed to precisely reveal the add‐on effects of oral CHM PSORI‐CM01. The experience and results obtained in this pilot study will contribute to refining the design of a future study, and assist the sample size calculation for the full‐scale RCT.

## CONSORT CHECKLIST

This study is reported following the checklist of items for Reporting Trials of Chinese Herbal Medicine Formulas (Supplementary file).

## CONFLICT OF INTEREST

The authors declare that there is no conflict of interest that could be perceived as prejudicing the impartiality of the research reported.

## ETHICS APPROVAL AND CONSENT TO PARTICIPATE

The trial's protocol has been previously published and was ethically approved by the RMIT University Human Research Ethics Committee and the Ethics Committee of Guangdong Provincial Hospital of Chinese Medicine. Written informed consent was obtained from all participants who were included in the eligibility screening.

## FUNDING INFORMATION

The study was jointly supported by the China‐Australia International Research Centre for Chinese Medicine (CAIRCCM) – a joint initiative of RMIT University, Australia and the Guangdong Provincial Academy of Chinese Medical Sciences, China, with additional funding support from the National Key Technology R&D Program for the 12th 5‐year Plan of Ministry of Science and Technology, China (grant number: 2013BAI02B03).

## AUTHOR CONTRIBUTIONS

Study design: Parker, AL Zhang, CS Zhang, Goodman, Wen, Lu, and Xue. Recruitment: Parker, Yan, Yao, Wu, and Deng. Screening and assessment of trial participants: Parker, Yan, Yao, Wu, and Deng. Statistical analyses of data: Parker, Zhang, and Zhang. Drafting of the manuscript: AL Zhang, Parker, CS Zhang, Wen, Goodman, Xue, and Lu. Revising of the manuscript: AL Zhang, CS Zhang, Lu, and Xue. Data collection: Parker, Yan, Yao, Wu, and Deng. All authors reviewed the manuscript draft prior to submission.

## AVAILABILITY OF DATA AND MATERIAL

Original data and other material will be available upon requests.
